# Suicidality and ADHD: which factors may influence the suicidal behavior?

**DOI:** 10.1192/j.eurpsy.2025.1628

**Published:** 2025-08-26

**Authors:** M. E. Riezzo, G. Longo, M. Servasi, S. Piergentili, R. Volgare, L. Orsolini, U. Volpe

**Affiliations:** 1 Unit of Clinical Psychiatry, Department of Clinical Neurosciences/DIMSC, Polytechnic University of Marche, Ancona, Italy

## Abstract

**Introduction:**

Patients with Attention-Deficit/Hyperactivity Disorder (ADHD) are at a higher risk of suicidal behavior. Impulsivity, emotional dysregulation, and co-existing mental health conditions contribute to this risk. Early identification and comprehensive support are vital in mitigating these issues.

**Objectives:**

The aims of our study are to characterize which factors promote suicide attempt in patient with ADHD.

**Methods:**

Our study is conducted on patients (>18 years) referred to the adult ADHD outpatient service of the Psychiatric Clinic of Ancona (Università Politecnica delle Marche, Italy). The Diagnostic Interview for ADHD in adults (DIVA 5.0) was used for diagnosing ADHD. The following rating scale were administered: Temperament Evaluation in Memphis, Pisa and San Diego (TEMPS-M), Coping Orientation to the Problems Experiences-new Italian version (COPE-NVI), Temperament and Character Inventory-Revised (TCI-R).

**Results:**

76% (n=170) of all screened patients were diagnosed with ADHD in adulthood. 7.3% (n=12) of patients with ADHD attempted suicide. A significant higher frequency of suicide attempts was observed in those with borderline personality disorder as a comorbidity (p=0.004), in those with other psychiatric comorbidities (p=0.031), in those who are receiving treatment from the pathological addiction outpatient service (p=0.011), and in those who use stimulants (p=0.018) or opioids (p=0.019). A multivariate linear regression was observed between number of suicidal attempt (R^2^=0.357; F(5,52)=5.779; p<0.001) and TCI-R transpersonal identification subscale (B=0.022; p=0.021), TCI-R harm avoidance subscale (B=-0.008; p=0.001), TCI-R impulsiveness subscale (B=0.023; p=0.012), TCI-R disorderliness subscale (B=-0.024; p=0.045) and COPE-NVI turn to religion subscale (B=-0.033; p=0.005). A logistic regression analysis was performed to ascertain the effects of all types of TCI-R subscale, on the likelihood of enacting suicide attempts. The logistic regression model was statistically significant, ▫▫2(1)=4.210, p=0.04. The model explained 14% (Nagelkerke R2) of the variance in patients with ADHD who committed suicide and correctly classified 90.3% of cases. Enacting a suicide attempt was significantly predicted TCI-R pure-hearted conscience subscale (exp(B)=0.843, p=0.062).

**Conclusions:**

Comorbidities, addiction treatment, substance use, and personality traits significantly influence the likelihood of suicidal behavior. In addition, it is highlighted that impulsivity, lack of responsibility and caution, those who feel a strong connection with nature and the universe, being rigid, not having transcendental-oriented coping strategies, and being opportunistic are factors promoting suicide in patients with ADHD.

**Disclosure of Interest:**

None Declared

